# The Multiple Roles of Trogocytosis in Immunity, the Nervous System, and Development

**DOI:** 10.1155/2021/1601565

**Published:** 2021-09-22

**Authors:** Eileen Uribe-Querol, Carlos Rosales

**Affiliations:** ^1^Laboratorio de Biología del Desarrollo, División de Estudios de Posgrado e Investigación, Facultad de Odontología, Universidad Nacional Autónoma de México, Mexico City 04510, Mexico; ^2^Departamento de Inmunología, Instituto de Investigaciones Biomédicas, Universidad Nacional Autónoma de México, Mexico City 04510, Mexico

## Abstract

Trogocytosis is a general biological process that involves one cell physically taking small parts of the membrane and other components from another cell. In trogocytosis, one cell seems to take little “bites” from another cell resulting in multiple outcomes from these cell-cell interactions. Trogocytosis was first described in protozoan parasites, which by taking pieces of host cells, kill them and cause tissue damage. Now, it is known that this process is also performed by cells of the immune system with important consequences such as cell communication and activation, elimination of microbial pathogens, and even control of cancer cells. More recently, trogocytosis has also been reported to occur in cells of the central nervous system and in various cells during development. Some of the molecules involved in phagocytosis also participate in trogocytosis. However, the molecular mechanisms that regulate trogocytosis are still a mystery. Elucidating these mechanisms is becoming a research area of much interest. For example, why neutrophils can engage trogocytosis to kill *Trichomonas vaginalis* parasites, but neutrophils use phagocytosis to eliminate already death parasites? Thus, trogocytosis is a significant process in normal physiology that multiple cells from different organisms use in various scenarios of health and disease. In this review, we present the basic principles known on the process of trogocytosis and discuss the importance in this process to host-pathogen interactions and to normal functions in the immune and nervous systems.

## 1. Introduction

Trogocytosis, a recently identified cellular process, is being recognized more and more as an important general biological activity for eukaryotic cell communication [1, 2]. During trogocytosis (from the Greek trogo-: nibble), one cell physically takes little pieces (“bites”) from another cell and ingests these pieces of cellular material. The process of trogocytosis has relevant consequences for both cells involved [3]. Trogocytosis is different from phagocytosis (from the Greek phago-: devour), where one cell ingests completely another cell [4, 5]. Trogocytosis is also different from other processes for cell-cell communication, such as nanotubes or exosomes. Trogocytosis is a rapid transfer process after direct contact between two living cells that passes on membrane fragments and intact proteins from one cell to the other [6–10]. Trogocytosis has been observed in various biological scenarios and has received different names, including partial phagocytosis, cell cannibalism, and cell nibbling [1, 11]. At present, it is not clear if all instances reported for trogocytosis utilize a unique conserved molecular mechanism or if they are different cellular processes. However, as more and more examples are being discovered and described, it seems that trogocytosis represents a universal conserved cellular process in eukaryotic biology. Trogocytosis was first described among amoebas. These eukaryotic organisms were observed to use trogocytosis for attacking and killing other cells [12–14]. Later, trogocytosis was detected between cells of the immune system [6, 7, 15]. Among immune cells, trogocytosis represents a gentle form of cell-cell contact, without causing cell death, that leads to important regulatory functions of the adaptive immune response [3, 16]. In the last few years, our awareness on trogocytosis has grown enormously due to reports showing that trogocytosis can be performed by many different cell types [17]. In the innate immune system, cells use trogocytosis for cell communication, elimination of pathogens, and control of tumor cells [8, 18, 19]. Also, some intracellular microorganisms use trogocytosis to transit from one cell to another [20, 21], and some protozoan parasites can kill host cells and evade the immune system by trogocytosis [14, 22]. In the central nervous system, trogocytosis is used for remodeling synaptic connections [23]. Finally, during development, embryonic cells also use trogocytosis for cell remodeling [11]. In this review, we will consider the known aspects of trogocytosis in these cell systems, and we will present some current questions on trogocytosis and possible future clinical applications.

## 2. Trogocytosis Is Used by Amoebas for Cell Killing

**Figure 1:**
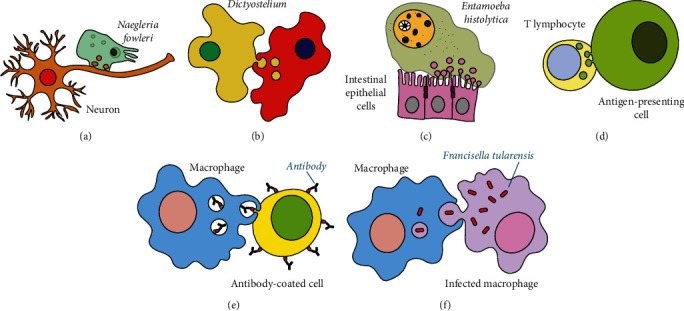
Examples of trogocytosis. (a) The “brain-eating” amoeba *Naegleria fowleri* can destroy neuron cells by taking (nibbling) little pieces off them. (b) The predatory slime mold *Dictyostelium caveatum* feeds upon other *Dictyostelium* species amoebas. (c) The intestinal parasitic amoeba *Entamoeba histolytica* actively nibbles pieces of live endothelial cells leading to cell lysis. (d) Immune cells use trogocytosis for cell communication. Antigens together with major histocompatibility complex (MHC) molecules can be transferred from antigen-presenting cells (APC) to T lymphocytes. After trogocytosis between immune cells, both cells continue alive. (e) Macrophages can perform trogocytosis to remove membrane antigens from antibody-coated cells. This form of trogocytosis has also been called antibody shaving. (f) Intracellular bacteria take advantage of trogocytosis to spread between cells. The bacteria *Francisella tularensis* infect and live within macrophages and can transfer from one cell to another through trogocytosis. In this process, the plasma membrane, cytoplasm, and live bacteria are transported from one infected macrophage to another.

Trogocytosis was first described among amoebas. These eukaryotic organisms were observed to use trogocytosis for attacking and killing other cells. The first example of trogocytosis was reported for the “brain-eating” amoeba *Naegleria fowleri*, which was shown to destroy mammalian cells by taking (nibbling) little pieces off them [12] (Figure 1(a)) (Table 1). *N. fowleri* cytopathic effect coincided with the accumulation of discrete particles containing mammalian cell components within the cytoplasm of amoebas. Thus, the term “trogocytosis” was proposed for describing this process [12]. The predatory slime mold, *Dictyostelium caveatum*, feeds upon other *Dictyostelium* species amoebas as opposed to bacteria (Figure 1(b)) (Table 1). This allows *D. caveatum* amoebas to increase in size by feeding upon cells the same size or even larger. The feeding mechanism was later reported to also involve nibbling pieces of prey cells [13]. More recently, it was found that the intestinal parasitic amoeba *Entamoeba histolytica* actively nibbles pieces of live epithelial cells leading to cell lysis [14] (Figure 1(c)). The cell damage induced by trogocytosis involves acidified lysosomes and cysteine proteinases [24, 25]. These proteinases seem to participate in trogocytosis but not in phagocytosis, suggesting different mechanisms for activating trogocytosis or phagocytosis in amoebas [25] (Table 1). In addition, to killing host endothelial cells for tissue invasion, *E. histolytica* also uses trogocytosis to evade the immune system [26]. *E. histolytica* is susceptible to complement-mediated lysis, but during trogocytosis, these amoebas can incorporate host cell membrane proteins, such as CD59 or DAF (decay accelerating factor), and display them on their membrane. In this manner, amoebas block the formation of the complement membrane attack complex (MAC) and become resistant to complement-mediated lysis [22, 27].

Although trogocytosis has been established as a mechanism used by amoebas to destroy other cells, it is important to remember that amoeba is just a morphological state that different eukaryotic organisms present. Amoebas in the examples mentioned above belong to separate species; thus, it seems that trogocytosis could be a universal process in eukaryotic cells. This idea is further supported on the various examples found in other cells as discussed next.

## 3. Cells of the Immune System Use Trogocytosis for Cell Communication

Trogocytosis was also detected in cells of the mammalian immune system. B cells were first observed to form a close contact “synapse” with antigens on the membrane of a target cell. B cells then nibble on the target cell and acquire little pieces of the antigen-containing membrane [6]. In this process, the B cell antigen receptor facilitates collecting antigen molecules into the synapse for their subsequent internalization, thereby improving antigen processing and presentation to T cells [6] (Table 1). Later, trogocytosis has been found to occur in T cells [7, 10, 15], natural killer (NK) cells [28], dendritic cells (DC) [29], macrophages [30], neutrophils [8], and basophils [31]. Opposite to amoebas, trogocytosis between immune cells leaves both cells alive. Thus, it is considered that it represents a gentle form of cell-cell communication [3].

Very early reports indicated that antigens (proteins) can be transferred from macrophages to lymphocytes [32]. Later, it was found that lymphocytes (T cells) acquire antigen together with major histocompatibility complex (MHC) molecules [15]. Several reports followed showing that antigens together with other plasma membrane proteins are transferred from antigen-presenting cells (APC) to lymphocytes [10, 33] (Figure 1(d)). Then, the term trogocytosis was used to describe this process, and it was also suggested that this exchange of molecules between cells could modulate the immune response [3]. Trogocytosis between APC and lymphocytes is a regulated process triggered by a selected set of surface molecules. Mainly, the T cell receptor (TCR) and the B cell receptor (BCR), together with some costimulatory molecules such as CD28, have been found to be important for initiating trogocytosis [33, 34].

Other examples of communication between immune cells via trogocytosis with consequences for the immune response involve several other cell types. Inhibition of CD4^+^ T cells by MHC class II-dressed NK cells resulted after NK cells interacted with DC and acquired MHC class II molecules from DC into their membranes [28]. NK cells concurrently acquired costimulatory molecules such as CD80 and CD86 from DC, but their expression did not reach functional levels. In consequence, the MHC class II-dressed NK cells inhibited DC-induced CD4^+^ T cell responses [28, 35]. Plasmacytoid dendritic cells (pDC) have limited capacity for phagocytosis, yet they are capable of presenting cell antigens to T cells. The explanation for this was found when it was discovered that human pDCs, although inefficient in the internalization of cell membrane fragments by phagocytosis, can efficiently acquire membrane portions from cancer cells via trogocytosis [29]. The transfer of the membrane also included intact human leukocyte antigen (HLA)-antigen (Ag) complexes, which could be efficiently recognized on pDC by tumor-specific CD8^+^ lymphocytes [29] (Table 1). Monocytes and macrophages were also found to perform trogocytosis of antibody-coated cells (Figure 1(e)). A process now reported as Fc gamma receptor- (Fc*γ*R-) mediated trogocytosis [36]. B cells treated with the anti-CD20 monoclonal antibody (mAb) rituximab were recognized, via Fc*γ*R, by RAW264.7 macrophages. This resulted in antigen removal from the B cell membrane [30]. Similarly, monocytes acquired CD8 *αβ* heterodimer molecules from anti-CD8 mAb-treated CD8^+^ lymphocytes [37]. The transfer of CD8 molecules required the expression of Fc*γ*RII on monocytes [37] (Table 1). Neutrophils are cells that also perform trogocytosis in different scenarios with multiple outcomes. First, it was reported that neutrophils could take membrane portions from monocytes (U937 cells) after cell-cell contact [8]. In addition, neutrophils could also acquire membrane segments from CD4^+^ lymphocytes. This exchange resulted in functional changes in the neutrophil, which showed enhanced phagocytosis and interleukin- (IL-) 8 production, and also delayed neutrophil apoptosis [8]. Molecules in the immunological synapse, including MHC class I and class II, the integrin LFA-1, and the chemokine receptor CXCR1, are exchanged among autologous neutrophils, CD4^+^ T cells, and U937 cells after cell-cell contact [8]. Membrane transfer from monocytes to neutrophils transduces survival and activation signals to enhance neutrophil functions, and it is dependent on actin polymerization, clathrin activation, and Fc receptors (Table 1). In contrast, membrane transfer from neutrophils to monocytes depends on MAP kinase and PKC signaling [8]. At the same time, lymphocytes also acquired membrane segments from the neutrophil, resulting in enhanced IL-2 production [8]. Also, membrane exchange between autologous neutrophils and CD4^+^ T cells led to transfer of lactoferrin from the neutrophil to the T cell resulting in suppressed interferon-gamma (IFN-*γ*) but enhanced IL-10 production [38]. These reports highlight the importance of bidirectional trogocytosis for modulating immune responses.

The molecular aspects of trogocytosis between immune cells are not clear, and there is even discrepancy in which cellular components are transferred. In the majority of reports, trogocytosis is described as the transfer of the membrane and membrane proteins, without intracellular components from one cell to another. In other reports, trogocytosis is described as the internalization of another cell intracellular components. Whether these two options of trogocytosis are part of the same process or two separate mechanisms remains to be resolved. The idea of only transfer of membrane components comes from studies using fluorescent membrane dyes and/or fluorescent proteins detected by flow cytometry. With this approach, cytoplasm transfer between cells was not detected [39]. Microscopy is an alternative method that offers more sensitivity and could detect cytoplasm transfer in addition to membrane transfer [14, 19], helping to resolve this issue. In addition to membrane transfer, recent reports support the idea that cytoplasmic components are also captured during trogocytosis. Neutrophils captured the cytoplasmic dye calcein from target cells after antibody-dependent cell-mediated cytotoxicity (ADCC) via trogocytosis [19]. Also, the cytoplasmic dye carboxyfluorescein succinimidyl ester (CFSE) was transferred from herpes simplex virus- (HSV-) infected monocyte-derived dendritic cells to plasmacytoid dendritic cells [40]. In addition, the transfer of bacteria between macrophages via trogocytosis (see later) was accompanied by transfer of calcein [20]. Future studies should resolve what components are passed from one cell to another, but in the case of immune cells, it seems that both intracellular components and membrane components are transferred during trogocytosis.

## 4. Intracellular Bacteria Take Advantage of Trogocytosis to Spread between Cells

The emerging view from the reports discussed above is that trogocytosis is indeed an important regulatory process in immunity, and as such, it is not surprising that some intracellular pathogens use trogocytosis to their advantage. The bacteria *Salmonella enterica* serovar *Typhimurium* and *Francisella tularensis* infect and live within macrophages and can transfer from one cell to another without going outside the macrophage. In this process, the plasma membrane, cytoplasm, and live bacteria were transported from one infected macrophage to another through trogocytosis [20, 21] (Figure 1(f)). After the process, bacteria were found within double-membrane vesicles composed by both the donor and recipient cell plasma membranes. Bacteria can escape from these vesicles using their type VI secretion system (T6SS) [21].

Other intracellular pathogens can also use trogocytosis to their advantage. Red blood cells infected with *Plasmodium falciparum* are trapped in brain microvessels causing cerebral malaria. In this case, infected red blood cells transferred membrane parts and *Plasmodium* antigens to endothelial cells in an actin-dependent manner similar to trogocytosis [41]. This was followed by internalization of red blood cells and opening of endothelial cell intercellular junctions, resulting in an enhanced inflammatory response with endothelial cell alterations associated with cerebral malaria [41]. Since other intracellular microorganisms also can transfer from cell to cell, it is likely that trogocytosis is a mechanism used in many other infections.

## 5. Neutrophils Use Trogocytosis to Kill Large Cells

The examples mentioned above of trogocytosis among cells of the immune system point to trogocytosis as a benign form of cell-cell communication. However, recent reports show that trogocytosis can also be used for cell killing by the immune system. Neutrophils implement trogocytosis to kill parasites and also sperm cells [18, 42].

**Figure 2:**
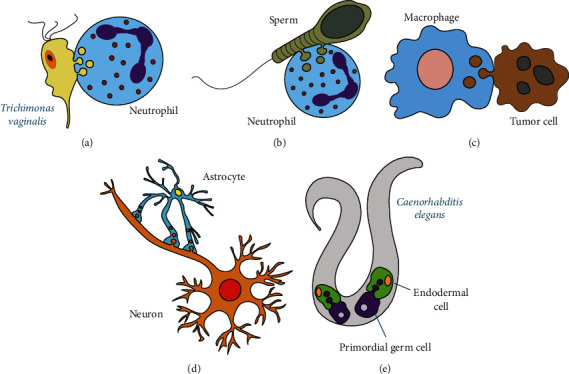
Examples of trogocytosis to kill large cells. (a) *Trichomonas vaginalis*, a large and highly motile parasite, is attacked by neutrophils which surround and take “bites” of the parasite membrane until the parasite dies. (b) In vaginal tissues, the excess of sperm is eliminated by neutrophils. Since sperm cells are too large to be phagocytosed, neutrophils take “bites” of the sperm membrane and quickly reduced their motility and viability. (c) Macrophages can perform trogocytosis to kill antibody-coated tumor cells. (d) Astrocytes which are the central nervous system glial cells responsible for regulating synaptic neuronal networks take pieces of axon projections containing mitochondria from the optic nerve neurons through trogocytosis. (e) During development, *Caenorhabditis elegans* primordial germ cells connect to endodermal precursor cells in the interior of the embryo. These primordial cells form lobes that are removed and digested by endodermal cells through trogocytosis. This form of trogocytosis has also been called cell cannibalism.

*Trichomonas vaginalis* are large and highly motile parasites responsible for a highly prevalent sexually transmitted infection. As an extracellular parasite, *T. vaginalis* adheres to epithelial cells to colonize the human host. In addition, the parasite interacts with cells of the innate immune system, mainly neutrophils [43]. The classical mechanisms recognized for neutrophil killing of pathogenic microorganisms include phagocytosis, degranulation of antimicrobial molecules, and the formation of neutrophil extracellular traps (NET) [44, 45]. Surprisingly, none of these mechanisms were found to participate in the killing of *T. vaginalis*. Yet, neutrophils rapidly killed this parasite in a dose-dependent and contact-dependent manner [18]. To achieve this end, neutrophils surround and take “bites” of the parasite membrane (Figure 2(a)) (Table 1). Interestingly, neutrophils performed trogocytosis only on live *T. vaginalis* and performed phagocytosis of dead parasites [18]. This behavior is similar to *E. histolytica*, which nibbles live human epithelial cells and performs phagocytosis of dead human cells (Figure 1(c)) [2, 14]. This suggests that trogocytosis may indeed be a conserved biological process.

In vaginal tissues, the excess of sperm is eliminated by neutrophils. Since sperm cells are too large to be phagocytosed, the mechanism of elimination was not clear. In a recent study, it was found that neutrophils could efficiently kill sperm cells in a contact-dependent and NET-independent manner [42] (Table 1). After contact, neutrophils took “bites” of sperm and quickly reduced their motility and viability [42] (Figure 2(b)). Neutrophil trogocytosis is then a novel process in the antimicrobial and immunomodulatory functions of neutrophils with relevant implications for health homeostasis and disease.

## 6. Trogocytosis for Control of Tumor Cells

In the fight against cancer, antibody therapy is one of the most important tools available today. In antibody therapy for cancer, the binding of antibodies to specific antigens on the membrane of tumor cells marks the cells as targets for several immune mechanisms. Antibodies can directly downregulate growth factor signals and arrest tumor growth or can lead to tumor cell death through various mechanisms, including complement-dependent cytotoxicity and cell-mediated cytotoxicity [36, 46]. However, the efficacy of antibodies can be reduced by the active removal of antigen-antibody complexes from the membrane of target cells. This event, originally called “antibody shaving,” is now recognized as active trogocytosis by macrophages and other cells (Figure 1(e)) [47]. The effect of trogocytosis has been well documented on the removal of the antigen CD20 and anti-CD20 mAbs (such as rituximab) from the membrane of lymphoma B cells, allowing the tumor cells to escape therapy (Table 1) [48, 49]. This form of trogocytosis is mediated by Fc*γ*R on the effector cell. Thus, in order to reduce the negative effect of trogocytosis, administration of lower doses of mAb has been proposed [50, 51]. In support of this approach, novel antibodies with higher affinity for Fc*γ*R at lower doses can still enhance ADCC activity and promote tumor cell death [52].

Despite the negative effect of trogocytosis, in which therapeutic antibodies are eliminated, recent reports also show that trogocytosis can indeed cause tumor cell death. By reducing the dose of the anti-CD20 mAb rituximab, macrophages presented less trogocytosis and as a result they had enhanced ADCC [50]. In live-cell time-lapse microscopy experiments, it was shown that neutrophils mediate trogocytosis of anti-CD20 mAb-opsonized leukemic B cells from patients with chronic lymphocytic leukemia (CLL). Trogocytosis was accompanied by loss of membrane CD20 from CLL B cells, leading to tumor cell death [53]. In addition, macrophages were also shown to kill HER2-positive breast cancer cells that were covered with the anti-HER2 mAb trastuzumab [54]. In this case, antibody engineering to increase its affinity for Fc*γ*R resulted in enhanced macrophage trogocytosis leading to tumor cell death [54]. In another study, using high-resolution *in vivo* imaging, macrophages in the liver (Kupffer cells) killed invariant natural killer (iNKT) cells through trogocytosis [55]. Kupffer cells ripped large fragments off crawling antibody-coated iNKT cells, causing iNKT cell death in liver sinusoids [55]. iNKT killing was dependent on Fc*γ*R and required high glycosylation of antibodies for strong binding of the antibody on iNKT cells to the Fc*γ*R on Kupffer cells [55]. In addition, the use of anti-HIV antibodies to mediate T cell killing was also shown to be mediated by trogocytosis [56]. CD4^+^ T cells expressing the viral gp120 protein were treated with anti-gp120 antibodies. These antibodies on the membrane of T cells also engaged Fc*γ*R on monocytes (THP-1 cells) and induced the transfer of membrane fragments from the T cell to the monocytes. In this exchange, THP-1 effector cells remained intact, while T cells lost viability gradually [56]. All these reports together strengthen the view that various kinds of macrophages can accomplish trogocytosis to kill tumor cells (Figure 2(c)).

In addition to macrophages, neutrophils can also kill tumor cells by ADCC. However, the mechanism for this cytotoxic effect is not clear. Recently, it was found that neutrophils can also kill tumor cells by antibody-mediated trogocytosis [19]. Killing required Fc*γ*R in cooperation with the integrin CD11b/CD18 and was potentiated by blocking CD47-SIRP*α* interactions. Thus, neutrophils perform ADCC of tumor cells via trogocytosis. The authors proposed the term “trogoptosis” to refer to trogocytosis resulting in cell death [19].

## 7. Trogocytosis for Cell Remodeling

In other cell types, outside the immune system, new examples of trogocytosis have been found. In the nervous system and during development, trogocytosis is used for cell remodeling. Microglia, the resident macrophages of the central nervous system, are highly motile glial cells that get rapidly activated during neurological diseases. They can produce inflammatory cytokines and phagocytose cell debris or damaged neurons. Microglia were also proposed to control synaptic pruning during neuronal circuit formation through controlled phagocytosis [57]. Hence, it is thought that improper communication between microglia and neurons leads to an excess of immature synaptic connections, because of defective phagocytosis of synapses by microglia [57]. However, a study using ex vivo preparations of the brain showed that microglia remodel synapses of neuronal cells by trogocytosis [23]. With the use of light sheet fluorescence microscopy to follow microglia-synapse interactions and of correlative light and electron microscopy (CLEM) to complete a 3D ultrastructural characterization, it was discovered that the small size of presynaptic material ingested by microglia was consistent with trogocytosis, rather than phagocytosis [23]. More recently, techniques like two-photon *in vivo* microscopy [58] were key for studying microglial remodeling of neuronal axons *in vivo* [59]. In this recent study, it was shown that microglia utilize trogocytosis for pruning retinal ganglion cell axons in the developing *Xenopus laevis* retinotectal circuit [59]. In addition, it was shown that microglia remodeling is important for proper behavioral response to dark and bright looming stimuli [59]. Hence, it is now recognized that microglial cells control development, maturation, and plasticity of neuronal ensembles by synaptic pruning, a process completed via trogocytosis [60].

In addition, astrocytes which are the central nervous system glial cells responsible for regulating synaptic neuronal networks [61] were also found to perform trogocytosis. Astrocytes constitutively internalize parts of neurons in the myelination transition zone of the optic nerve head [62]. Astrocytes took pieces of axon projections containing mitochondria from the optic nerve neurons (Figure 2(d)). These mitochondria were then degraded within lysosomes in the astrocyte [62]. Interestingly, similar deposits of degrading mitochondria were also found along neurites of the cerebral cortex, suggesting the possibility that trogocytosis may be a more general event in the nervous system [63]. Astrocytes are also important for shortening myelin segments at optic nerve neurons during *Xenopus laevis* metamorphosis. The capture by astrocytes of myelin projections involves some molecules related to phagocytosis, and it is similar to trogocytosis [64]. Together, these reports suggest that astrocytes perform trogocytosis on neighbor neurons to remodel synapses connections and to eliminate damaged organelles (Table 1).

During development, some embryonic cells have also been found to use trogocytosis for cell remodeling. This process has been observed during embryonic development in *Caenorhabditis elegans* and *X. laevis* [11, 65]. During *C. elegans* gastrulation, primordial germ cells connect to endodermal precursor cells in the interior of the embryo [66]. These primordial cells form lobes that are removed and digested by endodermal cells (Figure 2(e)) [11]. Endodermal cells were shown to actively remove and ingest the lobes from the primordial cell body in a process named “cell cannibalism,” which resembles trogocytosis. The result is that primordial germ cells are dramatically altered in size and mitochondrial content [11]. Similarly, during *X. laevis* gastrulation, endodermal cells were shown to migrate by amoeboid type movements. Cells presented protrusions, but in contrast to other instances of amoeboid migration, trailing edge retraction required “transendocytosis” by a neighboring cell [65]. This process led to formation of double-membraned vesicles in a cell and remodeling and retraction of the trailing edge in the endodermal cell [65]. Thus, transendocytosis is similar to trogocytosis. These examples show that endodermal cells are capable of performing trogocytosis for remodeling other cells during development (Table 1).

## 8. Molecular Mechanisms of Trogocytosis

We have seen that trogocytosis appears in multiple unicellular and pluricellular organisms with similar general properties. Yet, the molecular mechanisms involved are just beginning to be elucidated. At present, it is not clear whether all the examples of cells nibbling on other cells use a common conserved molecular process. However, some general principles seem to be required in all instances of trogocytosis.

In many reports, some of the molecules identified to participate in trogocytosis are also involved in the process of phagocytosis. Thus, it has been proposed that trogocytosis is simply a form of incomplete phagocytosis, where a cell takes a part of the target cell instead of ingesting the whole cell. However, when a phagocytic leukocyte such as macrophages or neutrophils cannot complete phagocytosis because the target particle is too big, it spreads over the target but it does not take in little pieces. This “frustrated phagocytosis” does not fit well with the active nibbling process of trogocytosis [67, 68]. In addition, trogocytosis requires a scission mechanism to remove parts of the plasma membrane from the target cell. This mechanism certainly requires generation of mechanical force, which is not observed in regular phagocytosis. Supporting this idea, some molecules involved in trogocytosis that participate in membrane binding and scission [11, 69, 70] are not normally involved in phagocytosis. In addition, trogocytosis or phagocytosis takes place in distinct conditions. For example, amoebas prefer to nibble on live cells, but take dead cells by phagocytosis [14]. Similarly, neutrophils attack live *T. vaginalis* by trogocytosis, while engage on phagocytosis of dead parasites [18]. Nonetheless, there is still much to learn about the differences between phagocytosis and trogocytosis. We will now describe the molecules known to participate in trogocytosis by some of the best studied trogocytic cells.

**Figure 3:**
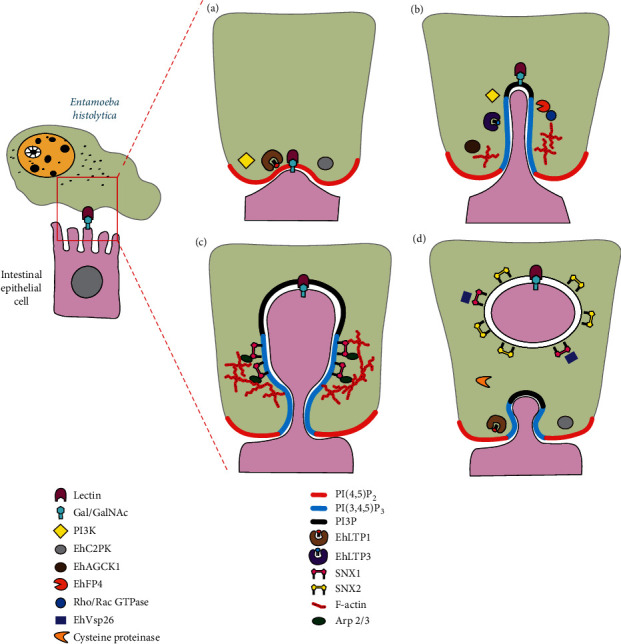
Molecular mechanisms of trogocytosis in *E. histolytica*. The molecular mechanisms involved in trogocytosis are just beginning to be elucidated. (a) The amoeba Gal/GalNAc (galactose and N-acetyl-D-galactosamine) lectin binds to glycoproteins on intestinal endothelial cells. In the amoeba, the enzymes PI3K (phosphatidylinositol 3-kinase) and EhC2PK (amoebic C2-kinase) get activated. The main phosphoinositide present in the resting plasma membrane is phosphatidylinositol 4,5-bisphosphate [PI(4,5)P_2_], which serves as a substrate for PI3K to generate phosphatidylinositol 3,4,5-trisphosphate [PI(3,4,5)P_3_]. Also, EhLTP1 (amoebic lipid transfer protein 1) (LTPs) is associated at the ligand attachment site at the initiation of trogocytosis. (b) PI(3,4,5)P_3_ is localized to the trogocytic cup as well as to the trogocytic tunnel. PI(3,4,5)P_3_ then recruits EhAGC kinases 1 (EhAGCK1) to the trogocytic cup, where it participates in regulating the formation of cytoskeletal structures that support the trogocytic tunnel. Also, PI(3,4,5)P_3_ recruits the FYVE domain-containing protein EhFP4 to the trogocytic tunnel. There, EhFP4 physically interacts with Rho/Rac small GTPases for controlling F-actin polymerization. Also, EhLTP3 (amoebic lipid transfer protein 3) is recruited onto the trogocytic tunnel at the intermediate stage of trogocytosis. (c) Also, phosphatidylinositol 3-phosphate (PI3P), another product of PI3K, accumulates to the distal end of the trogocytic cup. There, PI3P recruits EhSNX1 (amoebic sorting nexin SNX1), which specifically binds to Arp2/3 on the trogocytic tunnel to induce actin polymerization. (d) Finally, a trogosome is formed with a membrane enriched in PI3P, which also recruits EhSNX2 (amoebic sorting nexin SNX2). SNX1 on the trogosome membrane binds to the retromer component EhVps26, involved on transport of cysteine proteinases, which are also important for trogocytosis.

Trogocytosis was described in amoebas and it has been characterized in *E. histolytica.* As already mentioned, *E. histolytica* performs trogocytosis on live epithelial cells, but uses phagocytosis to ingest dead human cells [14]. Thus, these amoebas have become a good model for studying both processes. In *E. histolytica*, both processes are initiated by the galactose (Gal) and N-acetyl-D-galactosamine (GalNAc) lectin and involve the enzymes PI3K (phosphatidylinositol 3-kinase) and EhC2PK (amoebic C2-kinase) [14, 71] (Figure 3(a)). The main phosphoinositide present in the resting plasma membrane is PI(4,5)P_2_ (phosphatidylinositol 4,5-bisphosphate), which serves as a substrate for PI3K to generate PI(3,4,5)P_3_ (phosphatidylinositol 3,4,5-trisphosphate) [72]. PI(3,4,5)P_3_ is localized to the trogocytic cup as well as to the trogocytic tunnel (Figure 3(b)) [73]. PI(3,4,5)P_3_ then recruits EhAGC kinases 1 and 2 to the trogocytic cup. In particular, the kinase EhAGCK1 has been reported to be specific for trogocytosis [74]. EhAGCK1 participates in regulating the formation of cytoskeletal structures that support the trogocytic tunnel [74]. Also, PI(3,4,5)P_3_ recruits the FYVE (Fab1, YOTB1, Vac1, and EEA1) domain-containing protein EhFP4 to the trogocytic tunnel. There, EhFP4 physically interacts with Rho/Rac small GTPases for controlling actin polymerization (Figure 3(b)) [75]. In addition, another product of PI3K, the phosphatidylinositol 3-phosphate (PI3P), accumulates to the distal end of the trogocytic cup (Figure 3(c)). There, PI3P was shown to recruit the sorting nexins (SNXs) EhSNX1 and EhSNX2 to trogocytic structures. First, EhSNX1 was recruited to the trogocytic cup and trogocytic tunnel, and subsequently, EhSNX2 was recruited to trogosomes (Figures 3(c) and 3(d)) [76]. Furthermore, EhSNX1, but not EhSNX2, specifically bound to Arp2/3 on the tunnel-like structures and to EhVps26 on the trogosomes [76]. Thus, EhSNX1 participates in actin polymerization, that seems required to complete the formation of the trogosome (Figure 3(d)). Because, EhSNX2 gene silencing increased trogocytosis, it was suggested that EhSNX2 plays an inhibitory role in trogocytosis [76]. As seen from the examples above, the composition of lipids in the membranes regulates basic cellular processes such as cell adhesion, endocytosis, exocytosis, phagocytosis, and trogocytosis, by recruiting secondary effector molecules [77]. Consequently, nonvesicular lipid transport facilitated by lipid transfer proteins (LTPs) is also a key contributor to cell functions [78]. In *E. histolytica* amoebas, LTPs have been described as belonging to steroidogenic acute regulatory protein- (StAR-) related lipid transfer (START) proteins [79]. Among these LTPs, EhLTP1 and EhLTP3 were found to be involved in trogocytosis of live mammalian cells and phagocytosis of dead cells. EhLTP1 was associated at the ligand attachment site at the initiation of trogocytosis, followed by the recruitment of EhLTP3 onto the trogocytic tunnel at the intermediate stage of trogocytosis before the closure of the trogosome (Figures 3(a) and 3(b)) [80]. Interestingly, EhLTP1 is involved in trogocytosis and phagocytosis, while EhLTP3 is exclusively involved in trogocytosis of live host cells [80]. In addition, EhLTP1 participates on biosynthesis and secretion of cysteine proteinases [80]. The amoebic cysteine proteinases are also important for trogocytosis, since inhibition with E-64 caused a blockage of trogocytosis but not of phagocytosis (Figure 3(d)) [25]. Therefore, membrane lipid composition is also an important factor for recruiting effector molecules to the membranes and in this way influencing the decision of a cell to perform phagocytosis or trogocytosis. This is only a partial picture, and future research is required to characterize the molecular mechanisms in *E. histolytica* that are specific to trogocytosis.

**Figure 4:**
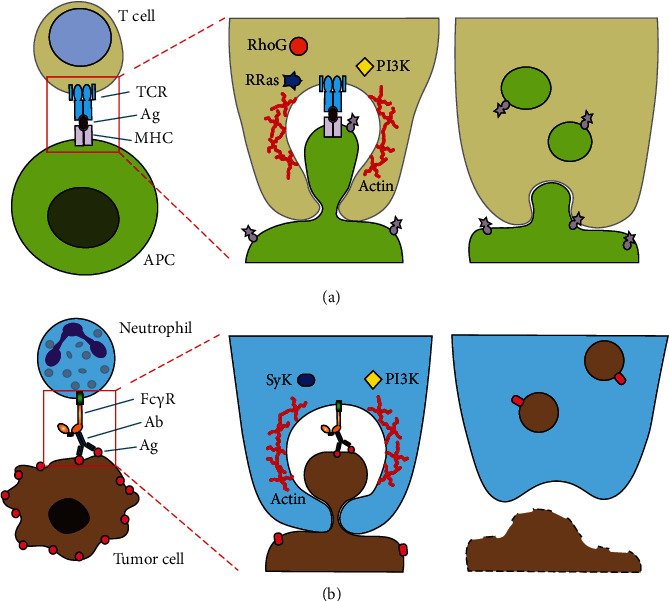
Molecular mechanisms of trogocytosis in immune cells. (a) T cell trogocytosis. T cell receptors (TCR) engage antigen (Ag) bound to major histocompatibility complex (MHC) molecules on the antigen-presenting cell (APC). In the T cell, the small GTPases RRas and RhoG, together with phosphatidylinositol 3-kinase (PI3K) and actin, participate in trogocytosis. Membrane proteins from the APC are transferred to the T cell. When the cells separate, both cells remain viable. (b) Neutrophils can kill tumor cells through trogocytosis. A tumor cell coated with antibodies (Ab) can be recognized by the neutrophil through its antibody Fc gamma receptors (Fc*γ*R). In the neutrophil, trogocytosis is activated by the participation of Syk (spleen tyrosine kinase), PI3K, and also actin. As a result of nibbling, the tumor cell gets killed.

In the case of cells of the immune system, trogocytosis is triggered by cell-cell contact mediated by receptor-ligand interactions. For lymphocytes (T cells, B cells, and NK cells), formation of an immunological synapse involving the antigen receptors TCR or BCR is the initial step of trogocytosis [9, 10, 34] (Figure 4(a)). Cytotoxic T cells (CTL) also acquire antigen and plasma membrane fragments from target cells in a TCR-dependent manner [7]. The antigen-MHC-TCR complexes are then internalized by the T cell [15, 33]. Similarly, two GTPases, RRas (TC21) and RhoG, have been identified to participate in T cell trogocytosis [70]. T cells used these molecules to promote uptake of MHC class II molecules from antigen-presenting cells. This uptake was also dependent on PI3K [70]. In several cases, participation of the actin cytoskeleton was reported to be important for T cell trogocytosis [10, 30, 33]. All of these molecules, except RRas, also participate in phagocytosis. Thus, much work is needed to elucidate the exact role of these molecules in trogocytosis.

In other immune cells, such as macrophages or neutrophils, antibody Fc*γ*R is involved in initiating trogocytosis [36, 50, 54, 55]. In this case, antibody-coated target cells (for example tumor cells) are recognized by Fc*γ*R, resulting in activation of ADCC mechanisms. In most cases, the killing process involves trogocytosis (Figure 4(b)). However, very little is known about the molecules signaling for trogocytosis after Fc*γ*R engagement. The signaling pathway used by these receptors to activate phagocytosis is fairly well described [81, 82]. After receptor aggregation, activation of Src family kinases phosphorylates the receptor on activating tyrosine residues within immunoreceptor tyrosine-based activation motifs (ITAMs). Next Syk (spleen tyrosine kinase) is recruited to these phosphorylated residues and in turn activates downstream enzymes, such as PI3K, PLC (phospholipase C), and PKC (protein kinase C). Also, small GTPases, such as Rac and Cdc42, get activated leading to reorganization of the actin cytoskeleton to form pseudopods around the particle to be ingested. The membrane protrusions close at the distal end forming a new phagosome that is internalized. This phagosome undergoes a maturation process and finally becomes a phagolysosome, where the ingested particle is degraded [83].

In the case of endodermal cell trogocytosis during *C. elegans* development, the molecules CED-10 (Rac), dynamin, and LST-4 (sorting nexin 9; SNX9) were identified as important players during trogocytosis of primordial germ cells [11]. Rac-induced actin together with dynamin and LST-4, was found around lobe necks and was required for lobe scission [11]. LST-4 is a multifunctional scaffold protein that coordinates membrane trafficking and remodeling [84], and dynamin has been shown to have a role in the effective scission of the phagosome from the plasma membrane [85]. Therefore, during trogocytosis, these proteins display a new role for active membrane excision and internalization of the removed cell components.

Finally, in the case of microglia trogocytosis, the activation signals are still unknown. However, the complement system has been implicated in this process. Overexpression of an endogenous membrane-bound complement inhibitory molecule prevented axonal pruning and trogocytosis. Thus, neurons exert local control on microglial trogocytosis and axonal pruning by expressing complement regulatory proteins [59]. Similarly, the amphibian regulator of complement activation 3 (aRCA3, a homolog of mammalian CD46) is a complement inhibitory molecule in *X. laevis*, and it is expressed in synapses of *X. laevis* retinal ganglion cells. Therefore, aRCA3 is also a good candidate molecule that participates in the mechanism for controlling trogocytosis [59].

## 9. Phagocytosis or Trogocytosis

Although some of the molecules involved in phagocytosis also seem to participate in trogocytosis, for example, actin, Syk, PI3K, and Rho/Rac small GTPases [19] (Figures 3 and 4), no much is known on how the cell might decide what process to activate after Gal/GalNAc lectin (in the case of *E. histolytica*) or Fc*γ*R engagement (in the case of leukocytes). One possibility is that the trogocytic cell “senses” the size of the target cell. A similar situation has been described in neutrophils in response to *Candida albicans*. When neutrophils encounter *C. albicans* in yeast form or single bacteria, they perform phagocytosis of these pathogens [86]. In contrast, neutrophils in the presence of *C. albicans* hyphae or extracellular aggregates of *Mycobacterium bovis* preferentially formed NET [86]. The pattern-recognition receptor dectin-1 acted as a sensor of microbe size and prevented NET release by downregulating the translocation of neutrophil elastase (NE) to the nucleus [86]. Other mechanisms for detecting cell size may involve the mechanosensing receptors PIEZO1 and TRPV4 (transient receptor potential vanilloid type 4) [87, 88]. PIEZO1 is a mechanically activated ion channel on immune cells that initiates an inflammatory response in the lungs [87], while TRPV4 is a mechanosensitive Ca^2+^ channel that regulates phagocytosis and mediates formation of ROS in neutrophils [88].

Another possibility is that activation of certain receptors is responsible for initiating either trogocytosis or phagocytosis. We mentioned that both amoebas and neutrophils prefer trogocytosis when confronted with live cells. Certain receptors could be responsible for distinguishing live from dead cells. In the case of apoptotic cells, phosphatidylserine receptors or scavenger receptors have been shown to be involved in recognition and induction of efferocytosis of apoptotic bodies [89, 90]. Besides, many cells die by necrosis and several novel receptors have been described that allow immune cells to also differentiate between live and necrotic cells [91]. All these receptors could potentially participate in initiating trogocytosis, and future research is required in this area to confirm their participation.

In the case of antibody-coated cells, immune Fc*γ*R-expressing cells may respond by displaying phagocytosis or trogocytosis. One possibility for the observed response is that activation of certain Fc*γ* receptors is responsible for initiating either trogocytosis or phagocytosis. Support for this idea comes from studies showing that in human neutrophils, particular cell responses are initiated by distinct Fc*γ*R [92]. So, for example, Fc*γ*RIIa is mainly a phagocytic receptor [93], while Fc*γ*RIIIb favors NET formation [94, 95]. Also, the fact that by reducing the dose of therapeutic antibodies, less trogocytosis was observed [50], and by increasing mAb affinity for Fc*γ*R resulted in enhanced macrophage trogocytosis [54], it is possible that certain antibodies interact better with certain Fc*γ* receptors. In addition, the use of novel chimeric antigen receptors (CARs) suggests that specific ligand receptor interactions may be responsible for initiating either trogocytosis or phagocytosis [96]. CARs are synthetic receptors that reprogram T cells to kill tumor cells, but can also be directed to induce phagocytosis. Among these new CARs for phagocytosis (CAR-P), those containing the cytosolic domains from Megf10 and the FcR*γ* chain efficiently triggered phagocytosis independently of their extracellular domain [96]. Together, these reports support the idea that specific receptor interactions will determine whether a cell initiates either trogocytosis or phagocytosis.

## 10. Perspectives

All examples presented above indicate that trogocytosis indeed is a very important process for many types of eukaryotic cells. However, trogocytosis is just beginning to be appreciated as a novel biological process with important implications for cell biology, development, immunity, and even clinical applications. One important issue that needs to be resolved is whether the different names, such as partial phagocytosis, cell cannibalism, cell nibbling, and transendocytosis found in the literature, are indeed various examples of the same process or trogocytosis is much more complex than we can imagine today [1, 11]. As we have seen, the outcome of trogocytosis varies considerably among cell types. In many cases, amoebas kill their target cell by trogocytosis [14, 97]. Yet, immune cells for the most part display a “gentle” type of trogocytosis that is relevant for cell-cell communication [6, 10, 28, 34]. Why amoebas kill their target cell, but immune cells do not? This question needs to be addressed in the near future, since resolving it would have important implications for immunity. Particularly, because immune cells can also kill target cells by trogocytosis [18, 19, 42, 54], it will be very helpful to understand how a neutrophil of a macrophage decides to just gently nibble the membrane of a neighbor immune cell to exchange information or vigorously take parts of the membrane of a parasite o tumor cell in order to kill it. With this information, one can imagine novel strategies to influence the immune system in future clinical applications to deliver more efficient responses for eliminating microbial pathogens and even controlling cancer cells.

In most reports, trogocytosis is described as a cell taking parts of the membrane from another cell [39]. Yet, in other reports, trogocytosis is described as the internalization of another cell intracellular components [14, 19]. Whether these two options of trogocytosis are parts of the same process or two separate mechanisms remains to be resolved. At least for immune cells, it seems that both intracellular components and membrane components are transferred during trogocytosis. This seems to be important for cell communication [1]. In the case of only transfer of the cell membrane, the target cell is more likely to be damaged and destroyed quickly [1]. Thus, finding what cellular components are taken by a trogocytic cell is important. However, solving this issue in the future will require the use of novel microscopic and labelling techniques.

In several cases, a trogocytic cell can differentiate between live and dead cells and initiate different processes, namely, trogocytosis or phagocytosis. Some receptors for detecting apoptotic or necrotic cells have been described and they can differentiate between live and dead cells [89–91]. However, there is not any information on whether these receptors could also initiate trogocytosis. It is possible that these receptors signal the trogocytic cell to engage the target cell, but it is also possible that these receptors only detect the type of target cell, and other, not yet described, receptors are the actual trogocytic initiators. Identifying bona fide trogocytic receptors would certainly make it easier to study this cell function and possible control it on different biological scenarios.

The majority of the molecules involved in trogocytosis also participate in phagocytosis. This implies that these two cellular responses are in many ways similar. However, the molecular mechanisms that regulate trogocytosis are still a mystery. Elucidating these mechanisms is becoming a research area of much interest. Two cellular models, the parasitic amoeba *E. histolytica* [22, 25] and neutrophils [8, 98], will certainly be used in future research to discover how each process is initiated and regulated. This research will certainly be complicated because we really do not know the exact function of many of the molecules identified to participate in both processes. Yet, novel technological advances and elegant experimental designs will provide important clues to understand how phagocytosis and trogocytosis are in fact different cellular responses.

Another interesting feature of trogocytosis is that it requires a scission mechanism to remove parts of the plasma membrane from the target cell. This mechanism certainly requires generation of a mechanical force, which is not observed in regular phagocytosis [11, 69, 70]. Discovering how the cell generates the force to rip apart another cell membrane will have important implications in cell biology. This knowledge would advance our understanding on how motile cells maintain their integrity while interacting with other cells.

These are some of the major questions that the field of trogocytosis is presenting us today. It is hard to imagine which of them are more important and should be addressed first. We believe that trogocytosis, as being a general biological process of many eukaryotic cells, would be investigated from many different angles in the near future. This is exciting, and it certainly should bring novel discoveries that hopefully make important contributions to immunology, cell biology, neurobiology, developmental biology, and even treatment of cancer.

## 11. Conclusions

Trogocytosis has moved from a curious mechanism shown by amoebas to kill other cells to a universal biological process with multiple implications for homeostasis. The immune system is tremendously affected by trogocytosis, and more and more we come to realize that this process regulates multiple effector functions. In the innate immune system, trogocytosis participates in elimination of invading pathogens and tumor cells, while in the adaptive immune system, trogocytosis activates or suppresses T cell responses. In addition, we have now several examples of this important biological process in regulating cell development and formation of neuronal connections in the nervous system. Trogocytosis, thus, appears to be a fundamental process in multiple eukaryotic organisms. Although some molecules involved in trogocytosis are also used for phagocytosis, these two processes are clearly different. Further investigation is required to better understand both cell responses and to be able to devise new potential therapeutic approaches both for infections and cancer. Certainly, new examples of trogocytosis will appear in the near future covering other aspects of cell biology.

## Figures and Tables

**Table 1:** Multiple cells perform trogocytosis.

Trogocytic cell	Target cell	Mechanism involved in trogocytosis	Ref.
Trogocytosis is used by amoebas for cell killing			
*Naegleria fowleri*	Neurons	Amoebas destroy mammalian cells by taking (nibbling) little pieces off them.	[12, 99, 100]
*Dictyostelium caveatum*	Other *Dictyostelium* species	*D. caveatum* are able to eat amoebas larger than themselves by nibbling pieces of the cells until they are small enough to be ingested. *D. caveatum* amoebas have the capacity to ingest amoebae of other *Dictyostelium* species, but do not attack each other. *Dictyostelium* amoebae faced with starvation trigger a developmental program during which many cells aggregate and form fruiting bodies that consist of a ball of spores held aloft by a thin stalk.	[101] [102, 103]
*Entamoeba histolytica*	Live intestinal epithelial cells	Amoebas attach to host cell glycoproteins containing galactose (Gal) or N-acetyl-galactosamine (GalNAc) via their Gal/GalNAc lectin. Upon attachment, trogocytosis signaling involves PI-3K and EhC2PK, both of which promote actin polymerization. Cell damage induced by trogocytosis involves acidified lysosomes and cysteine proteinases. *E. histolytica* is susceptible to complement-mediated lysis, but during trogocytosis, amoebas can incorporate host cell membrane proteins, such as CD59, and become resistant to complement-mediated lysis.	[14, 97], [24, 25], [22, 27]
Cells of the immune system use trogocytosis for cell communication			
B cells	Cells with cognate antigens	B cells form a close contact “synapse” with antigens on the membrane of a target cell. B cells then nibble on the target cell and acquire little pieces of antigen-containing membrane.	[6]
T cells B cells	Antigen-presenting cells (APC) and lymphocytes	Lymphocytes acquire antigen together with major histocompatibility complex (MHC) molecules. CD28 is important for initiating trogocytosis.	[10, 15, 33, 34]
NK cells	Dendritic cells	Acquisition of MHC class II molecules and costimulatory molecules such as CD80 and CD86. These MHC class II-dressed NK cells inhibited DC-induced CD4^+^ T cell responses.	[28, 35]
Plasmacytoid dendritic cells (pDC)	Cancer cells	Acquisition of intact MHC-antigen (Ag) complexes which could be efficiently recognized by tumor-specific CD8^+^ T lymphocytes.	[29]
Macrophages	Antibody-coated cells	Fc gamma receptor- (Fc*γ*R-) mediated trogocytosis removes antibodies “antibody shaving” from cells, allowing tumor cells to escape therapy.	[30, 36, 37, 47–49]
Neutrophil	Monocytes T and B cells	Acquisition of MHC class I and class II, the integrin LFA-1, and the chemokine receptor CXCR1. Trogocytosis activates survival and activation signals to enhance neutrophil functions. Signaling involves actin polymerization, clathrin activation, and Fc receptors.	[8, 98]
Neutrophil	*Trichomonas vaginalis*	Neutrophils surround and take “bites” of the parasite membrane.	[18]
Neutrophil	Sperm cells	Neutrophils took “bites” of sperm and quickly reduced their motility and viability.	[42]
Macrophage	Bacteria-infected macrophage	Live bacteria were transported from one infected macrophage to another. Bacteria then escape from trogocytic vesicles using their type VI secretion system (T6SS).	[20, 21]
Macrophage	Tumor cell	Reducing the dose of mAb, macrophages presented less trogocytosis and enhanced ADCC, leading to tumor cell death. Antibody engineering to increase its affinity for Fc*γ*R resulted in enhanced trogocytosis leading to tumor cell death.	[50, 53, 54]
Neutrophil	Tumor cell	ADCC of tumor cells via trogocytosis required Fc*γ*R in cooperation with the integrin CD11b/CD18 and was potentiated by blocking CD47-SIRP*α* interactions.	[19]
Trogocytosis is used for cell remodeling			
Microglia	Neurons	Microglia control synaptic pruning during neuronal circuit formation through presynaptic trogocytosis. Microglia utilize trogocytosis for pruning retinal ganglion cell axons in the developing *Xenopus laevis* retinotectal circuit.	[23, 57, 59]
Astrocytes	Neurons	Astrocytes took pieces of axon projections containing mitochondria from the optic nerve neurons. Astrocytes took myelin projections at optic nerve neurons during *Xenopus laevis* metamorphosis.	[62, 64]
*Caenorhabditis elegans* endodermal cells	Primordial germ cells	Endodermal cells remove and ingest the lobes from the primordial cell body in a process named “cell cannibalism,” which resembles trogocytosis.	[11]
*Xenopus laevis* endodermal cells	Other endodermal cells	During metamorphosis, trailing edge retraction of migrating endodermal cells required trogocytosis by a neighboring cell.	[65]
